# Toxicity and mechanism of bis(2-ethylhexyl) phthalate in premature ovarian failure: A network toxicology prediction and molecular docking analysis

**DOI:** 10.1097/MD.0000000000043404

**Published:** 2025-07-18

**Authors:** Shaoming Ge, Qian Li, Yuexing Li, Chuhan Cao, Yuman Shi, Yunzhi Chen

**Affiliations:** a School of Basic Medicine, Guizhou University of Traditional Chinese Medicine, Guiyang, Guizhou Province, China.

**Keywords:** DEHP, estrogen, molecular docking, network toxicology, premature ovarian failure

## Abstract

This study investigates the mechanism by which bis(2-ethylhexyl) phthalate (DEHP) induces premature ovarian failure (POF) using network toxicology analysis and molecular docking methods. The potential toxic targets of DEHP were identified using databases such as STITCH and SwissTargetPrediction. Disease-related targets associated with POF were retrieved from the GeneCards and OMIM databases. The overlapping targets related to DEHP-induced POF were identified by intersecting the 2 datasets. Furthermore, the protein–protein interaction network was constructed using the STRING database and visualized through Cytoscape software to identify key targets. Gene ontology enrichment and Kyoto Encyclopedia of Genes and Genomes pathway analyses were performed on the overlapping targets using the DAVID database. Finally, molecular docking simulations were performed using AutoDock Vina to validate the findings. A total of 116 intersection targets associated with DEHP-induced POF were identified. Among these, 6 key core targets were screened: caspase 3, b-cell lymphoma 2 (BCL2), matrix metalloproteinase 9, peroxisome proliferator-activated receptor gamma, BCL2L1, and cyclin D1. Gene ontology analysis revealed that these targets are primarily involved in response to xenobiotic stimulus, assembly of cyclin-dependent protein kinase holoenzyme complex, and BH domain binding. Kyoto Encyclopedia of Genes and Genomes analysis indicated that these targets are predominantly associated with critical signaling pathways, including the p53 signaling pathway and apoptosis pathways. Additionally, molecular docking studies demonstrated low binding energies between DEHP and the core target proteins, supporting their potential role in the pathophysiology of DEHP-induced POF. This study indicates that caspase 3, BCL2, matrix metalloproteinase 9, peroxisome proliferator-activated receptor gamma, BCL2L1, and cyclin D1 might be potential toxic targets of DEHP-induced POF. It reveals the potential molecular mechanisms underlying DEHP-induced POF, emphasizing the synergistic roles of the p53 signaling pathway and apoptosis in its pathogenesis. These insights provide a novel theoretical framework and identify potential therapeutic targets for the prevention and management of POF.

## 1. Introduction

Premature ovarian failure (POF) is a complex endocrine disorder that affects women of reproductive age, characterized by a significant decline in ovarian function before the age of 40.^[1]^ This condition is generally irreversible and leads to premature menopause. The primary clinical features include nonphysiological amenorrhea, infertility, elevated follicle-stimulating hormone levels exceeding 40 U/L, and reduced estrogen levels.^[2]^ These symptoms are frequently accompanied by perimenopausal manifestations such as hot flashes, night sweats, diminished libido, irritability, and osteoporosis. Systematic reviews and meta-analyses estimate the global prevalence of POF at approximately 3.5%, with a concerning trend of increasing incidence among younger populations.^[3]^ Moreover, studies indicate that the natural pregnancy rate in patients with POF is low, ranging from 5% to 10%.^[4]^ The pathogenesis of POF is multifactorial, encompassing genetic predispositions, environmental and social factors, autoimmune conditions, psychological and psychiatric disorders, as well as exposure to chemotherapy agents.^[5]^ While sex hormone replacement therapy is commonly used to manage POF, its efficacy in restoring ovarian function and inducing ovulation remains limited. Additionally, HRT is associated with notable side effects, including an increased risk of endometrial cancer, breast cancer, and other adverse outcomes.^[6]^ Given the significant emotional and physical burden of POF, particularly for women with reproductive aspirations, early prevention, and intervention strategies are essential.

Bis(2-ethylhexyl) phthalate (DEHP) is the most extensively utilized plasticizer in the production of plastic products and is prevalent in various household items, such as children’s toys, food packaging, mineral water bottles, personal care products, and medical devices. DEHP does not form a stable bond with polymers like polyvinyl chloride, facilitating its migration from plastic products into the environment and food.^[7]^ Consequently, DEHP can enter the human body via multiple routes, including dermal absorption, ingestion, inhalation, and even transfusions. Moreover, as a lipophilic compound, DEHP tends to accumulate in the sebaceous layer of the skin’s epidermis and adipose tissue.^[8]^ Chronic exposure to DEHP can lead to significant accumulation in the body, resulting in adverse effects on the reproductive system, endocrine system, liver, and kidneys in both humans and animals.^[9–11]^ In response to these concerns, China’s hygienic standard for the use of additives in food containers and packaging materials restricts DEHP use to packaging materials intended for nonfatty foods, setting a maximum migration limit of 1.5 mg/kg. Similarly, the European Union and the United States mandate that DEHP content in plastics used for children’s toys and childcare articles must not exceed 0.1%. Furthermore, the European Food Safety Authority has established a tolerable daily intake for DEHP in food contact materials at 0.05 mg/kg body weight.^[12]^ Despite these regulatory measures, studies have reported significantly elevated concentrations of DEHP in human biological samples, including urine, blood, milk, semen, and follicular fluid, indicating potential health risks.^[13]^

POF is primarily attributed to follicular dysfunction resulting from follicular atresia, a process closely associated with the apoptosis of ovarian granulosa cells.^[14,15]^ Studies in mice exposed to DEHP have demonstrated alterations in the distribution and morphology of ovarian granulosa cells, a significant reduction in oocyte numbers, and an increase in follicular destruction and atresia.^[16,17]^ Moreover, DEHP exposure has been linked to impaired sperm quality in men and reproductive toxicity in women.^[18]^ A study by Park et al indicated that DEHP exhibits antiestrogenic effects, disrupting estrogen production pathways.^[19]^ The consequent decrease in estrogen levels is a hallmark of POF, while dysregulation of ovarian granulosa cell populations represents a key pathological feature of this condition. Based on these observations, we hypothesize that DEHP may adversely affect ovarian function, potentially contributing to the development of POF.

To verify the hypothesis, we utilized network toxicology for analysis. Network toxicology, an approach derived from network pharmacology, integrates concepts and methodologies from both network pharmacology and network biology. This method enables the prediction of toxic targets of compounds from a toxicological perspective and facilitates the analysis of their toxicity through a network model that links “toxicity – compound – gene – protein.”^[20]^ In this study, we applied network toxicology to investigate the toxic pathways associated with DEHP-induced POF, predict the underlying molecular mechanisms of DEHP toxicity, and provide insights for early prevention strategies of POF.

## 2. Materials and methods

### 2.1. Prediction and screening of the correlated targets of DEHP

The chemical structure and SMILES notation of DEHP were initially retrieved from the PubChem database (https://pubchem.ncbi.nlm.nih.gov/). Using the obtained SMILES notation, potential pathogenic targets associated with DEHP were predicted via the STITCH 5.0 database (http://stitch.embl.de/) and SwissTargetPrediction database (http://www.swisstargetprediction.ch/). Subsequently, the toxicity-related targets of DEHP were reconstructed by integrating the predicted targets from these databases.

### 2.2. Screening of POF related targets

To identify potential targets associated with POF, an extensive search was conducted using the keyword “POF” across multiple authoritative online databases, including GeneCards (https://www.genecards.org/), OMIM (https://www.omim.org/), DrugBank (https://go.drugbank.com/), and PharmGKB (https://www.pharmgkb.org/). The disease targets for POF were identified by consolidating and de-duplicating the results obtained from these databases.

### 2.3. Construction of protein–protein interaction network and identification of core targets

To elucidate the relationship between the target genes associated with DEHP and POF, we utilized the online Venn Diagram platform (https://bioinfogp.cnb.csic.es/tools/venny/) to generate a Venn diagram. The intersecting genes identified from this analysis were considered potential targets for DEHP-induced POF. These intersecting genes were then submitted to the STRING database (https://cn.string-db.org/) for protein–protein interaction (PPI) network analysis. For this analysis, we restricted the species to “Homo sapiens,” set the “Minimum required interaction score” to “Medium Confidence > 0.4,” and opted to “Hide disconnected nodes in the network.” The resulting PPI network data in TSV format was subsequently imported into Cytoscape software for further visual analysis. Topological analysis of the network was conducted using the CytoNCA tool, which calculated various topological parameters such as degree, betweenness, closeness, and local average connectivity for each node. Nodes with a degree greater than twice the median value, along with other parameters exceeding the median threshold, were selected as core targets. The resulting core target network was visualized to facilitate further interpretation.

### 2.4. Gene ontology (GO) and Kyoto Encyclopedia of Genes and Genomes (KEGG) enrichment analyses of potential targets

To elucidate the biological functions and pathways associated with the potential targets of DEHP-induced POF, GO, and KEGG enrichment analyses were performed using the DAVID database (https://davidbioinformatics.nih.gov/). The species was specified as “Homo sapiens,” and a significance threshold of “*P* < .05” was applied for screening. The results were ranked in ascending order based on their *P*-values. The top 10 GO terms and the top 20 KEGG pathways with the lowest *P*-values were selected and visualized using bar and bubble charts generated via the bioinformatics online mapping platform (https://www.bioinformatics.com.cn/).

### 2.5. Molecular docking validation of DEHP with core targets

The 6 core targets with the highest Degree values were identified and selected as protein receptors for molecular docking, using DEHP as the ligand. The 3D structures of these targets were retrieved from the RCSB Protein Data Bank (https://www.rcsb.org/). Preprocessing of the target proteins was conducted using PyMOL software, which involved removing water molecules and native ligands, followed by adding hydrogen atoms to optimize the protein structures. The chemical structure of DEHP was obtained from the PubChem database and further optimized through energy minimization using Chem 3D software. Subsequently, AutoDock Tools-1.5.7 was employed to add hydrogen atoms and configure rotatable bonds for DEHP. The processed protein structures and DEHP were then imported into AutoDock Vina via AutoDock Tools to calculate binding affinity and binding energy. The docking results were visualized through PyMOL software. Binding energies less than −5.0 kcal/mol were considered indicative of good binding activity, while values below −7.0 kcal/mol suggested strong binding activity.^[21]^

## 3. Results

### 3.1. Targets screening of DEHP-induced POF

**Figure 1.**
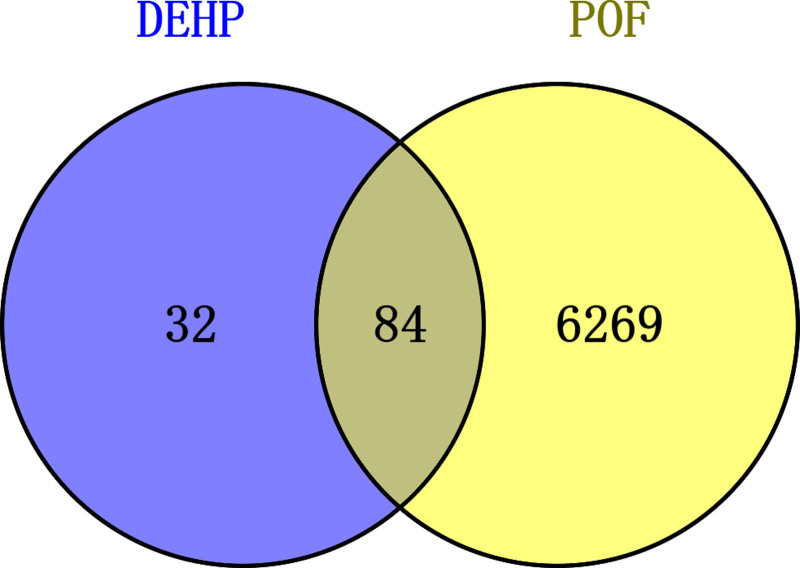
Venn diagram illustrating the overlapping targets of DEHP and POF. DEHP = bis(2-ethylhexyl) phthalate, POF = premature ovarian failure.

In this study, a total of 116 unique potential DEHP-related targets were identified from the STITCH and SwissTargetPrediction databases after removing duplicate entries. Furthermore, 6353 POF-associated targets were retrieved from the GeneCards, OMIM, DrugBank, and PharmGKB databases. By utilizing the online Venn Diagram platform, we identified 84 overlapping targets shared between DEHP and POF, which may represent potential pathogenic targets involved in DEHP-induced POF (Fig. 1; Table 1).

**Table 1:** Potential pathogenic targets involved in DEHP-induced POF.

No.	Gene names	Description	Uniprot ID
1	CASP3	Caspase-3	P42574
2	CYP3A4	Cytochrome P450 family 3 subfamily A member 4	P08684
3	PPARG	Peroxisome proliferator-activated receptor gamma	P37231
4	PPARA	Peroxisome proliferator-activated receptor alpha	Q07869
5	IL10	Interleukin 10	P22301
6	PLA2G4A	Phospholipase A2 group IVA	P47712
7	ATF3	Activating transcription factor 3	P18847
8	CA2	Carbonic anhydrase 2	P00918
9	MYOD1	Myogenic differentiation 1	P15172
10	AR	Androgen receptor	P10275
11	PRKCD	Protein kinase C delta	Q05655
12	PTPN1	Protein tyrosine phosphatase non-receptor type 1	P18031
13	PRKCA	Protein kinase C alpha	P17252
14	PTPN2	Protein tyrosine phosphatase non-receptor type 2	P17706
15	CTSK	Cathepsin K	P43235
16	CTSL	Cathepsin L	P07711
17	CTSB	Cathepsin B	P07858
18	FKBP1A	FKBP prolyl isomerase 1A	P62942
19	PDE10A	Phosphodiesterase 10A	Q9Y233
20	GRM2	Glutamate metabotropic receptor 2	Q14416
21	CCND1	Cyclin D1	P24385
22	CDK1	Cyclin dependent kinase 1	P06493
23	CCNE1	Cyclin E1	P24864
24	MAPK14	Mitogen-activated protein kinase 14	Q16539
25	TSPO	Translocator protein	B1AH88
26	PTGER1	Prostaglandin E receptor 1	P34995
27	GABRB3	Gamma-aminobutyric acid type A receptor subunit beta3	P28472
28	BCL2	BCL2 apoptosis regulator	P10415
29	GPBAR1	G protein-coupled bile acid receptor 1	Q8TDU6
30	ACE	Angiotensin I converting enzyme	P12821
31	KCNA5	Potassium voltage-gated channel subfamily A member 5	P22460
32	TNFRSF1A	TNF receptor superfamily member 1A	P19438
33	SCN10A	Sodium voltage-gated channel alpha subunit 10	Q9Y5Y9
34	MEN1	Menin 1	O00255
35	TRPC3	Transient receptor potential cation channel subfamily C member 3	Q13507
36	ELANE	Elastase, neutrophil expressed	P08246
37	TRPV4	Transient receptor potential cation channel subfamily V member 4	Q9HBA0
38	TRPV1	Transient receptor potential cation channel subfamily V member 1	Q8NER1
39	TRPA1	Transient receptor potential cation channel subfamily A member 1	O75762
40	SLC2A1	Solute carrier family 2 member 1	P11166
41	GRM5	Glutamate metabotropic receptor 5	P41594
42	ADAM17	ADAM metallopeptidase domain 17	P78536
43	CMA1	Chymase 1	P23946
44	PTGDR2	Prostaglandin D2 receptor 2	Q9Y5Y4
45	SLC2A3	Solute carrier family 2 member 3	P11169
46	SLC2A2	Solute carrier family 2 member 2	P11168
47	ADORA1	Adenosine A1 receptor	P30542
48	ADORA2A	Adenosine A2a receptor	P29274
49	MMP9	Matrix metallopeptidase 9	P14780
50	MMP2	Matrix metallopeptidase 2	P08253
51	MMP7	Matrix metallopeptidase 7	P09237
52	PABPC1	Poly(A) binding protein cytoplasmic 1	P11940
53	ABL1	ABL proto-oncogene 1, non-receptor tyrosine kinase	P00519
54	CCND3	Cyclin D3	P30281
55	CAPN1	Calpain 1	P07384
56	SCN2A	Sodium voltage-gated channel alpha subunit 2	Q99250
57	PTGES	Prostaglandin E synthase	O14684
58	PTGFR	Prostaglandin F receptor	P43088
59	AGTR1	Angiotensin II receptor type 1	P30556
60	TERT	Telomerase reverse transcriptase	O14746
61	KCNN4	Potassium calcium-activated channel subfamily N member 4	O15554
62	VCP	Valosin containing protein	P55072
63	MTNR1B	Melatonin receptor 1B	P49286
64	SLC6A9	Solute carrier family 6 member 9	P48067
65	PDE4A	Phosphodiesterase 4A	P27815
66	PDE4B	Phosphodiesterase 4B	Q07343
67	BAD	BCL2 associated agonist of cell death	Q92934
68	PIK3CA	Phosphatidylinositol-4,5-bisphosphate 3-kinase catalytic subunit alpha	P42336
69	MCL1	MCL1 apoptosis regulator, BCL2 family member	Q07820
70	BCL2L1	BCL2 like 1	Q07817
71	BCL2L2	BCL2 like 2	Q92843
72	BCL2A1	BCL2 related protein A1	Q16548
73	CRHR1	Corticotropin releasing hormone receptor 1	P34998
74	MAPK8	Mitogen-activated protein kinase 8	P45983
75	APP	Amyloid beta precursor protein	P05067
76	SCARB1	Scavenger receptor class B member 1	Q8WTV0
77	TDO2	Tryptophan 2,3-dioxygenase	P48775
78	TACR3	Tachykinin receptor 3	P29371
79	IDO1	Indoleamine 2,3-dioxygenase 1	P14902
80	CCNB1	Cyclin B1	P14635
81	CDK2	Cyclin dependent kinase 2	P24941
82	CDK4	Cyclin dependent kinase 4	P11802
83	CDK5	Cyclin dependent kinase 5	Q00535
84	CCND2	Cyclin D2	P30279

DEHP = bis(2-ethylhexyl) phthalate, POF = premature ovarian failure.

### 3.2. Construction of PPI networks and core target identification

**Figure 2.**
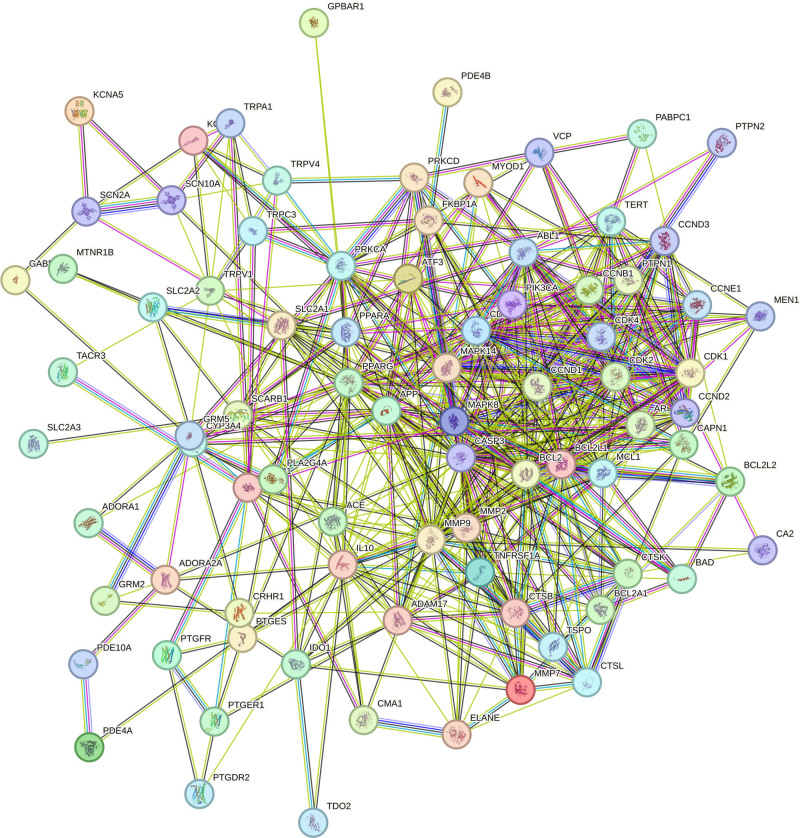
PPI network of pathogenic targets in DEHP-induced POF. DEHP = bis(2-ethylhexyl) phthalate, POF = premature ovarian failure, PPI = protein–protein interaction.

**Figure 3.**
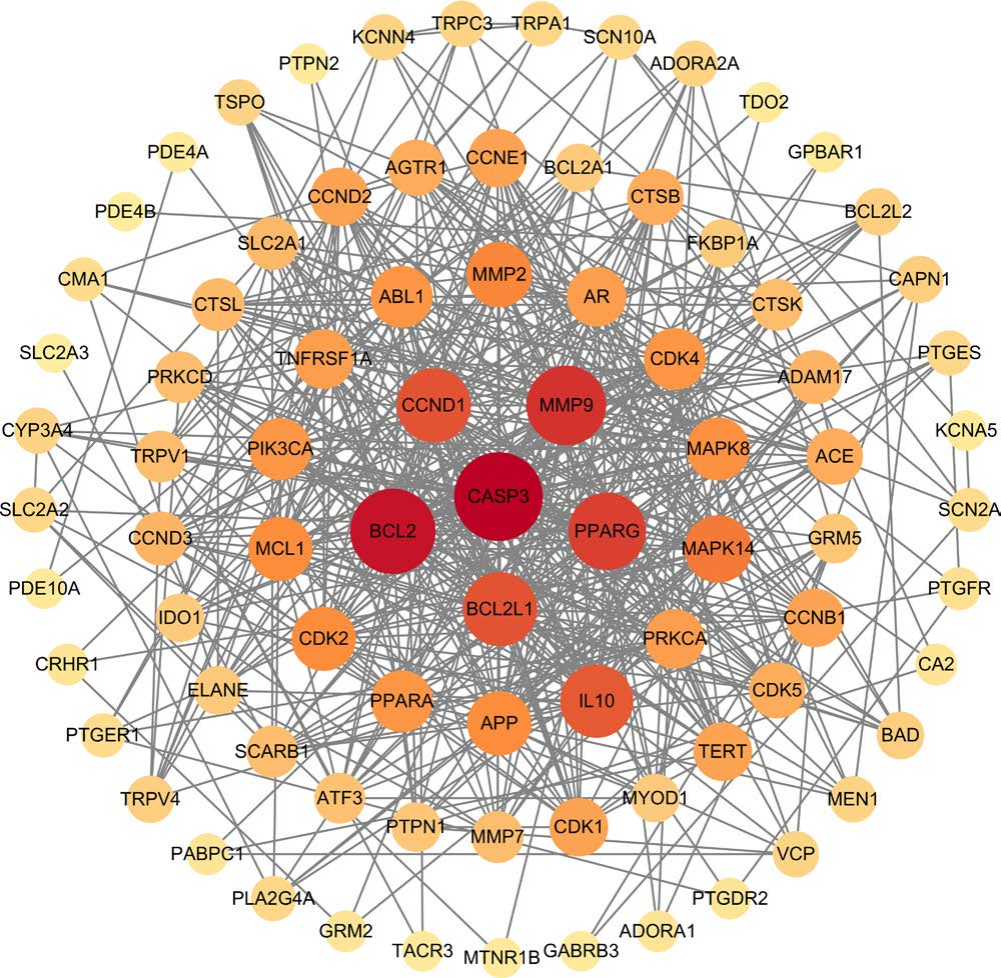
Visualization of the PPI Network. PPI = protein–protein interaction.

**Figure 4.**
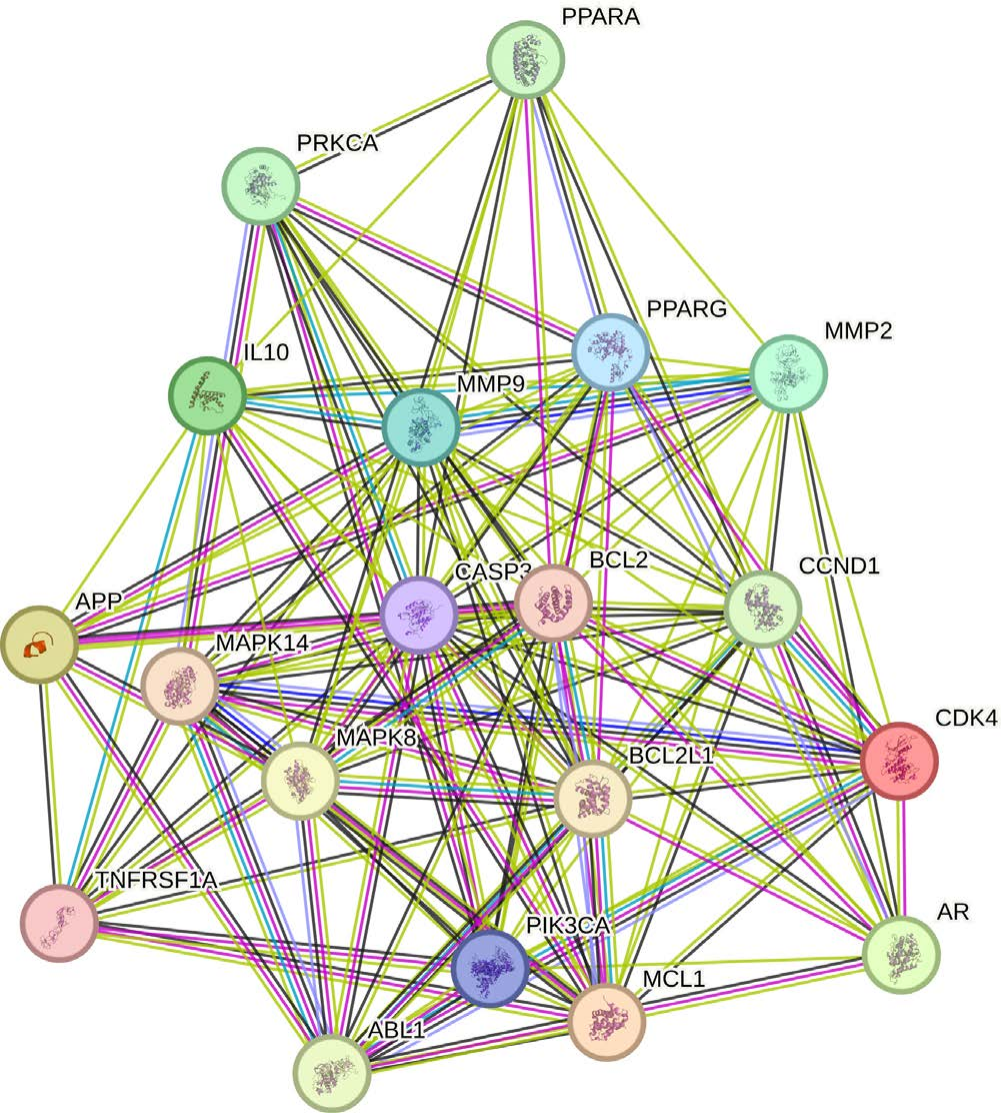
Core target PPI. PPI = protein–protein interaction.

The target genes identified at the intersection were used to construct a PPI network via the STRING database (Fig. 2). The PPI network was subsequently analyzed and visualized using Cytoscape 3.9.1 software (Fig. 3). This network comprises 83 nodes and 524 edges, resulting in an average node degree of 12.627. Based on topological parameters including degree, betweenness, closeness, and local average connectivity, 19 core targets were pinpointed (Fig. 4 and Table 2). Among these, 6 core targets with the highest degree scores – Caspase-3 (CASP3), BCL2, matrix metalloproteinase 9 (MMP9), peroxisome proliferator-activated receptor gamma (PPARG), BCL2L1, and cyclin D1 (CCND1) – were selected for further molecular docking validation.

**Table 2:** Topological parameters of the core targets in PPI.

No.	Gene names	Degree	Betweenness	Closeness	LAC
1	CASP3	45	750.0504	0.6666667	14.844444
2	BCL2	42	395.30554	0.63076925	15.428572
3	MMP9	37	450.0445	0.6119403	13.72973
4	PPARG	35	735.8645	0.6074074	10.6285715
5	BCL2L1	32	208.45564	0.58156025	14.375
6	CCND1	32	228.86649	0.57746476	14.5625
7	IL10	31	770.6158	0.58992803	9.67742
8	MAPK14	26	188.02782	0.5503356	11.769231
9	MMP2	24	85.04753	0.5466667	12.25
10	MCL1	23	56.511917	0.50931674	13.478261
11	APP	23	419.1805	0.5694444	7.7391305
12	MAPK8	22	82.30007	0.54304636	12.090909
13	PPARA	21	216.80977	0.5394737	7.2380953
14	PIK3CA	21	62.32853	0.50931674	12.190476
15	CDK4	21	32.13044	0.50617284	13.619047
16	ABL1	21	142.75204	0.5030675	11.047619
17	PRKCA	19	300.4732	0.53246754	6.4210525
18	TNFRSF1A	19	131.93518	0.50931674	8.842105
19	AR	19	60.13855	0.49101797	10.315789

PPI = protein–protein interaction.

### 3.3. GO and KEGG enrichment analysis

GO and KEGG enrichment analyses were conducted on 84 potential targets using the DAVID database, with a significance threshold of *P* < .05 for target selection. The GO analysis identified a total of 299 GO terms, which were categorized into 192 biological processes (BP), 52 cellular components, and 55 molecular functions. Furthermore, KEGG pathway analysis revealed significant enrichment in 99 signaling pathways.

**Figure 5.**
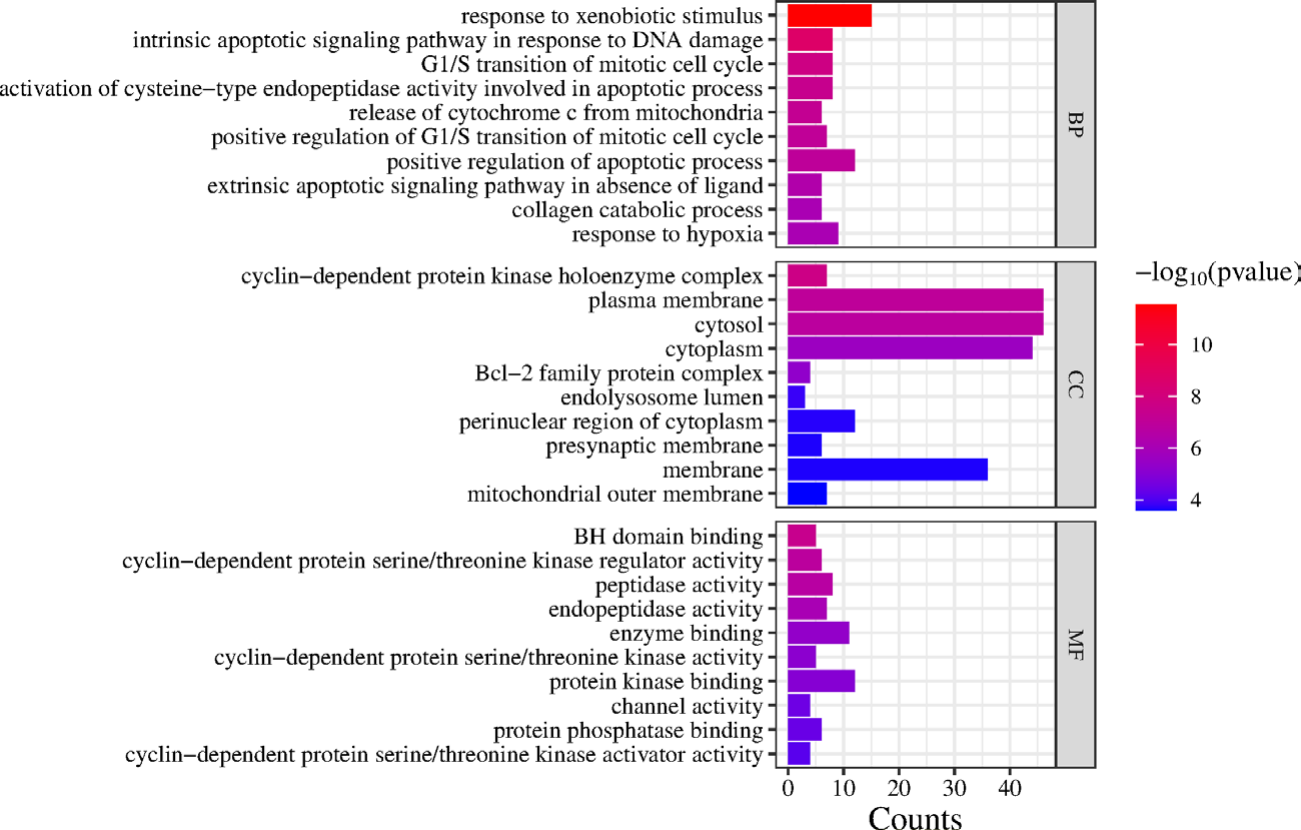
GO enrichment analysis. GO = gene ontology.

**Figure 6.**
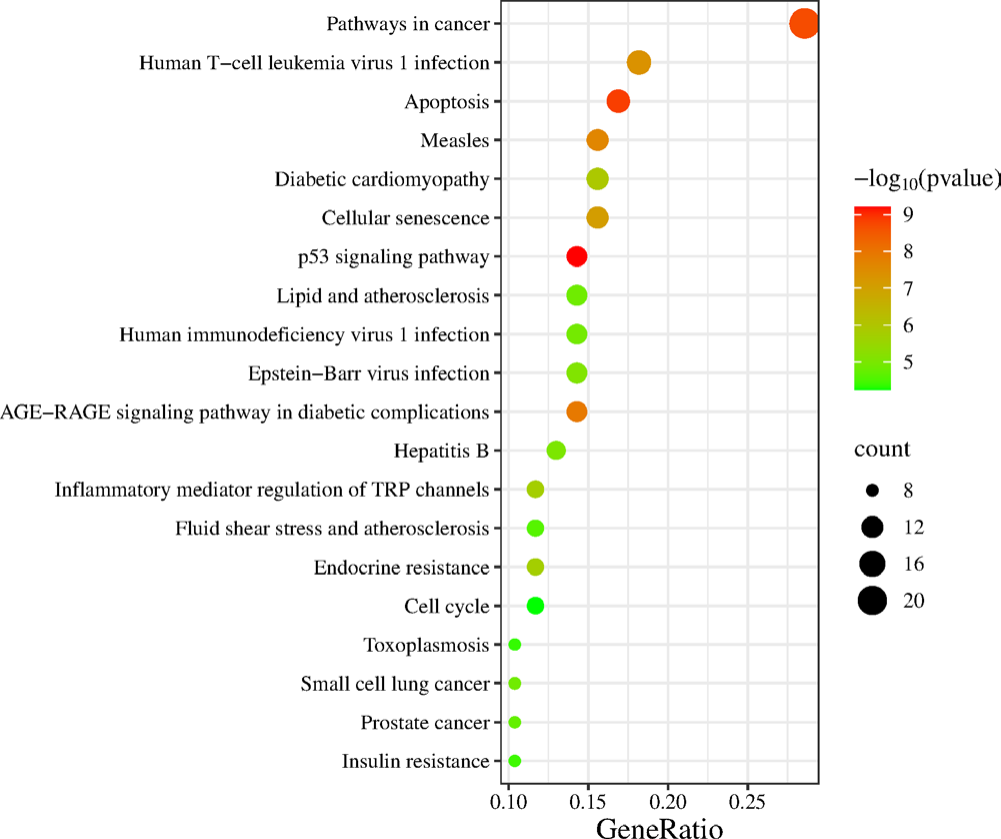
KEGG enrichment analysis. KEGG = Kyoto Encyclopedia of Genes and Genomes.

The GO analysis indicated that the primary BP included response to xenobiotic stimuli, intrinsic apoptotic signaling in response to DNA damage, G1/S transition during the mitotic cell cycle, and cytochrome *c* release from mitochondria. The cellular components prominently enriched included cyclin-dependent protein kinase holoenzyme complex, BCL2 family protein complex, and the mitochondrial outer membrane. The molecular functions predominantly featured BH domain binding, cyclin-dependent protein serine/threonine kinase regulator activity, and peptidase activity (Fig. 5). Additionally, KEGG pathway analysis demonstrated significant enrichment in several key pathways, such as the p53 signaling pathway, and apoptosis pathways (Fig. 6).

### 3.4. Molecular docking

**Figure 7.**
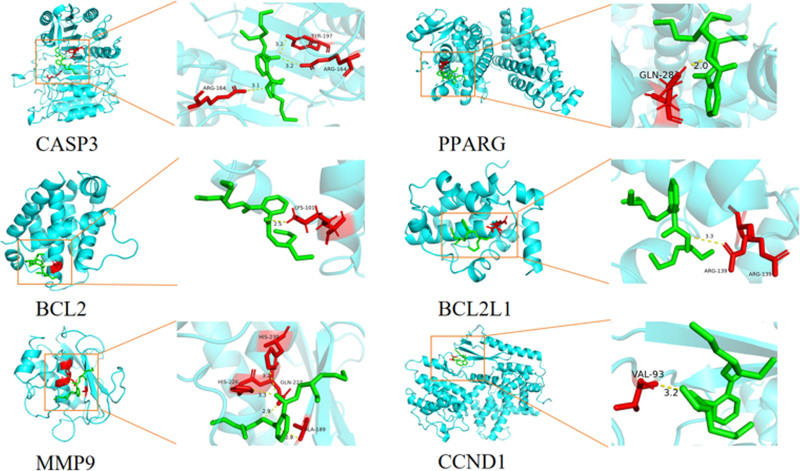
Molecular docking diagram illustrating the interaction between DEHP and core target. DEHP = bis(2-ethylhexyl) phthalate.

To validate the predicted targets, molecular docking simulations were conducted using AutoDock 1.5.7 to evaluate the binding energy between DEHP and the core target proteins. The simulation results revealed that the binding energy for all core target proteins interacting with DEHP was consistently below −5.0 kcal/mol, indicating a robust binding energy. This suggests a significant interaction between DEHP and the core targets associated with POF (Table 3). The most favorable docking configurations were selected for visualization (Fig. 7).

**Table 3:** The binding energy of DEHP and coreprotein receptors.

Gene names	PDB ID	Binding energy (kcal/mol)
CASP3	6BG4	−7.1
BCL2	5UUK	−6.3
MMP9	6ESM	−7.7
PPARG	8SC9	−7.4
BCL2L1	3SP7	−7.8
CCND1	6P8E	−6.3

## 4. Discussion

POF is a multifactorial endocrine disorder characterized by the premature decline in ovarian function and abnormalities in hormonal secretion. The etiology of POF is complex, encompassing genetic predisposition, environmental factors, and both physical and psychological health conditions. The onset of POF is typically marked by a reduction in the primordial follicular pool, accelerated follicular atresia, and a decline in ovarian reserve.^[22]^ In recent years, the prevalence of POF has increased, influenced by factors such as the rapid pace of modern life, environmental degradation due to industrialization, and the growing participation of women in the workforce. Patients with POF often experience a range of physical and psychological consequences, including amenorrhea, infertility, and symptoms associated with perimenopause.

Ovarian granulosa cells, which are pluripotent cells surrounding the oocytes, play a critical role in the development of primary follicles. These cells are indispensable for estrogen synthesis, supporting normal ovarian function, and providing a favorable microenvironment during folliculogenesis and ovulation.^[23,24]^ Furthermore, ovarian granulosa cells facilitate oocyte development through bidirectional signaling and nutrient transport to the surrounding oocytes via gap junctions.^[25]^ The crosstalk and interactions between granulosa cells and oocytes are essential for proper follicle formation and successful ovulation.^[26]^ Studies have shown that the decline in ovarian function is closely associated with the reduced biological capacity of atretic follicles.^[27]^ Specifically, apoptosis of ovarian granulosa cells accelerates follicular atresia, depletes the ovarian reserve, and contributes to the onset of POF.^[28]^

DEHP is commonly detected in human body fluids, and its primary metabolite, mono(2-ethylhexyl) phthalate, is found at the highest concentration in follicular fluid.^[29]^ Research has demonstrated that concurrent exposure to polystyrene microplastics and DEHP significantly increases the production of reactive oxygen species, thereby intensifying oxidative stress.^[30]^ This heightened oxidative stress induces DNA damage, compromising the integrity of ovarian granulosa cells in mouse follicles and ultimately leading to follicular fragmentation and atresia. In this study, we utilized network toxicology to explore the potential pathogenesis of POF induced by DEHP. Through this approach, we identified several key molecular targets associated with DEHP-induced POF, including CASP3, BCL2, MMP9, PPARG, BCL2L1, and CCND1.

CASP3 functions as a terminal effector in both the intrinsic and extrinsic apoptotic pathways. It is activated by initiator caspases, such as CASP8 and CASP9, leading to the cleavage of key intracellular substrates and ultimately initiating the apoptotic cascade.^[31]^ B-cell lymphoma 2 (BCL2), a well-established antiapoptotic protein, plays a crucial role in the mitochondrial apoptotic pathway by inhibiting the activation of the caspase cascade.^[32]^ Exposure to DEHP has been demonstrated to upregulate the expression of apoptosis-related genes, including CASP3 and Bax, while concurrently inducing oxidative stress in ovarian granulosa cells.^[33]^ MMP9, a member of the matrix metalloproteinase family, is critically involved in the degradation of the extracellular matrix and connective tissue during follicular development and ovulation, thereby facilitating tissue remodeling.^[34]^ Overexpression of MMP9 can result in excessive degradation of the extracellular matrix, impair the maturation of ovarian granulosa cells, induce aberrant apoptosis, and contribute to follicular atresia.^[35]^ PPARG, a member of the nuclear hormone receptor superfamily, regulates diverse physiological processes such as apoptosis, inflammation, and estrogen synthesis. PPARG competes for binding to estrogen response elements in ovarian granulosa cells, thereby inhibiting the expression of estrogen-responsive genes and modulating estrogen’s physiological effects.^[36]^ As a peroxisome proliferator, mono(2-ethylhexyl) phthalate, the active metabolite of DEHP, has been shown to reduce aromatase expression in ovarian granulosa cells via PPARG activation, consequently inhibiting the conversion of androgens to estrogens.^[37]^ CCND1, a key regulator of the cell cycle, facilitates cellular progression through various phases of the cell cycle.^[38]^ Research has demonstrated that in mice with POF, CCND1 axis expression is significantly downregulated, leading to cell cycle arrest in ovarian granulosa cells. This arrest impairs cell proliferation and differentiation, ultimately triggering apoptosis and contributing to ovarian dysfunction, thereby accelerating POF progression.^[39]^

GO analysis revealed that the induction of POF by DEHP was closely associated with several key BP, including response to xenobiotic stimulus, the intrinsic apoptotic signaling pathway in response to DNA damage, the G1/S transition during the mitotic cell cycle, the release of cytochrome *c* from mitochondria, the cyclin-dependent protein kinase holoenzyme complex, the BCL2 family protein complex, and the mitochondrial outer membrane. Exogenous stimuli, such as DEHP exposure, can provoke oxidative stress in the organism, resulting in DNA damage and subsequently triggering the mitochondria-dependent intrinsic apoptotic pathway. Upon activation of the endogenous apoptotic pathways, pro-apoptotic members of the BCL2 family, including Bax and Bak, translocate to the mitochondrial outer membrane. This process leads to the formation of permeability transition pores, which disrupt mitochondrial membrane integrity and facilitate the release of pro-apoptotic factors, such as cytochrome *c*, into the cytoplasm. This release ultimately activates CASP3, a key effector protease that drives apoptosis through the cleavage of essential cellular substrates.^[40]^ The cyclin-dependent protein kinase (CDK) holoenzyme complex consists of cyclin and CDK subunits. It is a catalytically active complex formed upon activation of cyclin-dependent kinases, such as CDK4/CDK6. This complex plays a vital role in regulating the transition from the G1 phase to the S phase of the mitotic cell cycle, ensuring proper cell cycle progression. By facilitating this transition, the cyclin-CDK complex helps to prevent apoptosis that may otherwise result from cell cycle arrest, thus maintaining cellular viability and function.

The KEGG pathway enrichment analysis mainly highlighted the p53 signaling pathway and apoptosis-related pathways. The p53 gene, a well-established tumor suppressor, plays a pivotal role in regulating apoptosis through its interaction with promoters of apoptosis-related genes that contain p53 response elements. Activation of the p53 signaling pathway initiates the apoptotic cascade, ultimately leading to cell death.^[4]^ A study by Sun Bo et al demonstrated that exosomes derived from bone marrow mesenchymal stem cells alleviated symptoms in mouse models of POF. This intervention significantly reduced follicular atresia and granulosa cell apoptosis, while concurrently suppressing the expression of CASP3 and p53.^[41]^ Furthermore, molecular docking simulations revealed that DEHP exhibited binding energy less than −5 kcal/mol with key core targets, particularly showing strong interactions (binding energy < −7 kcal/mol) with CASP3, MMP9, PPARG, and BCL2L1.

## 5. Conclusions

In this study, network toxicology and molecular docking approaches were utilized to explore the potential targets and mechanisms underlying DEHP-induced POF. Our results indicate that CASP3, BCL2, MMP9, PPARG, BCL2L1, and CCND1 may serve as critical pathogenic targets in DEHP-induced POF. Furthermore, our findings suggest that DEHP likely exerts its toxic effects via activation of the p53 signaling pathway and apoptosis-related signaling cascades.

## Acknowledgments

We express our gratitude to all the databases used in the article and all the participants involved in our study.

## Author contributions

**Data curation:** Chuhan Cao, Yuman Shi.

**Funding acquisition:** Yunzhi Chen.

**Project administration:** Yunzhi Chen.

**Visualization:** Yuexing Li.

**Writing – original draft:** Shaoming Ge.

**Writing – review & editing:** Qian Li.
